# Translational regulation of APOBEC3G mRNA by Vif requires its 5′UTR and contributes to restoring HIV-1 infectivity

**DOI:** 10.1038/srep39507

**Published:** 2016-12-20

**Authors:** Santiago Guerrero, Camille Libre, Julien Batisse, Gaëlle Mercenne, Delphine Richer, Géraldine Laumond, Thomas Decoville, Christiane Moog, Roland Marquet, Jean-Christophe Paillart

**Affiliations:** 1 Université de Strasbourg, CNRS, Architecture et Réactivité de l′ARN, UPR 9002, IBMC-15 rue René Descartes, F-67000, Strasbourg, France; 2 Université de Strasbourg, INSERM, UMR 1109, Laboratoire d’ImmunoRhumatologie Moléculaire, Fédération de Médecine Translationnelle de Strasbourg (FMTS), Institut de Virologie, 3 rue Koeberlé, F-67000 Strasbourg, France

## Abstract

The essential HIV-1 viral infectivity factor (Vif) allows productive infection of non-permissive cells expressing cytidine deaminases APOBEC3G (A3G) and A3F by decreasing their cellular level, and preventing their incorporation into virions. Unlike the Vif-induced degradation of A3G, the functional role of the inhibition of A3G translation by Vif remained unclear. Here, we show that two stem-loop structures within the 5′-untranslated region of A3G mRNA are crucial for translation inhibition by Vif in cells, and most Vif alleles neutralize A3G translation efficiently. Interestingly, K26R mutation in Vif abolishes degradation of A3G by the proteasome but has no effect at the translational level, indicating these two pathways are independent. These two mechanisms, proteasomal degradation and translational inhibition, similarly contribute to decrease the cellular level of A3G by Vif and to prevent its incorporation into virions. Importantly, inhibition of A3G translation is sufficient to partially restore viral infectivity in the absence of proteosomal degradation. These findings demonstrate that HIV-1 has evolved redundant mechanisms to specifically inhibit the potent antiviral activity of A3G.

The viral infectivity factor (Vif) of human immunodeficiency virus type 1 (HIV-1) and related lentiviruses neutralizes members of the APOBEC3 (Apolipoprotein B mRNA editing enzyme, catalytic polypeptide-like 3) family of restriction factors, allowing productive viral replication in non-permissive cells expressing these factors^1^,^2^,^3^,^4^. Among these cytidine deaminases, APOBEC3G (here referred to as A3G), A3F, A3D and A3H efficiently block HIV-1 replication after entry^5^,^6^,^7^,^8^,^9^,^10^. In the absence of HIV-1 Vif, A3G is efficiently incorporated into progeny virions through interactions with the nucleocapsid domain of Pr55^Gag^ and/or RNAs^11^,^12^,^13^,^14^,^15^. Once a new infection is initiated, the incorporated A3G molecules deaminate deoxycytidine to deoxyuridine in minus strand viral DNA during reverse transcription, resulting in hypermutation of the viral genome. As a result, the HIV-1 proviral DNA is no longer functional or/and rapidly degraded^6^,^16^,^17^,^18^. Additionally, deaminase-independent activity of A3G/3F has been shown to inhibit the accumulation of HIV-1 reverse transcription products and provirus integration^7^,^19^,^20^,^21^,^22^. Both cytidine deamination and inhibition of reverse transcription contribute to the antiviral activity of endogenous A3G/A3F proteins in CD4+ T cells^23^.

Vif reduces the intracellular A3G levels and its incorporation into viral particles by several mechanisms^2^,^4^,^24^. First, it has now been well documented that Vif recruits an E3 ubiquitin ligase complex that polyubiquitinates A3G/A3F proteins and targets them for proteasomal degradation^3^,^4^,^25^,^26^. Vif is composed of several highly conserved motifs that form discontinuous surfaces, so that Vif can accommodate all A3 proteins and the E3 ligase^27^,^28^. Moreover, the cellular transcription factor CBF-β was identified as a cofactor associated with the ubiquitin-like Cul5/Rbx2/EloBC (CRL5) complex and extensive interactions are involved in maintaining the binding of Vif and CBF-β^29^,^30^,^31^. CBF-β has been shown to stabilize Vif, thus allowing efficient degradation of A3G and increasing viral infectivity^32^,^33^,^34^,^35^. Second, it has been proposed that Vif could reduce the intracellular level of A3G by affecting its translation^36^,^37^. However, these studies were performed using expression vectors lacking the authentic 5′ and 3′ untranslated regions (UTRs) of A3G mRNA, which could play key role(s) in A3G translation^38^,^39^, and they thus may not faithfully recapitulate events occurring with endogenous A3G mRNA. Indeed, an *in vitro* translation study highlighted the importance of the 5′-UTRs of A3G mRNA in the inhibition of A3G translation by Vif^40^,^41^. However, the relative importance of the translational inhibition of A3G by Vif, compared to the well-documented A3G degradation, and its impact on viral infectivity remained to be established.

Here, we used several A3G mRNA expression plasmids mutated in their UTRs, with and without inhibitors of A3G degradation by the proteasome. Our data show that two stem-loop structures in the 5′-UTR of A3G mRNA are required for translational inhibition by Vif. The property of Vif to inhibit the translation of A3G is common to a large variety of Vif alleles and was also demonstrated in HIV-1 chronically infected H9 cells. In addition, we identified a mutation in Vif, K26R, which abolishes degradation of A3G by the proteasome but has no effect on the translational repression of A3G, demonstrating that these two pathways are independent. These two mechanisms contribute to the decrease of the intracellular level of A3G by Vif and to the subsequent A3G incorporation into virions. Importantly, the inhibition of A3G translation by Vif is sufficient to partially restore viral infectivity in A3G expressing cells in the absence of proteasomal degradation. These findings demonstrate that HIV-1 has evolved several redundant mechanisms to specifically inhibit the potent antiviral activity of A3G proteins.

## Results

### Vif impairs translation of A3G mRNA

**Figure 1:**
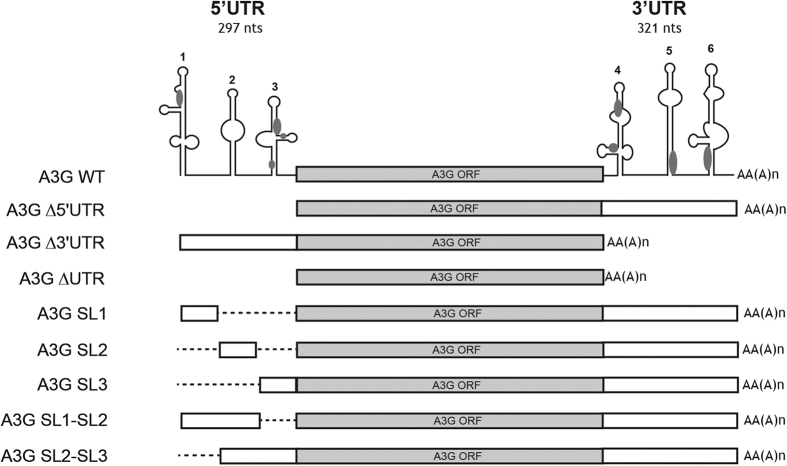
Schematic representation of A3G constructs used in this study. Wild-type authentic A3G mRNA and mutants deleted from their 5′, 3′ or 5′ and 3′-UTRs are represented. Secondary structures of the 5′- and 3′UTRs of wild-type A3G mRNA are also indicated with high affinity binding sites for Vif depicted in grey. Dotted lines represent the deletions.

**Figure 2:**
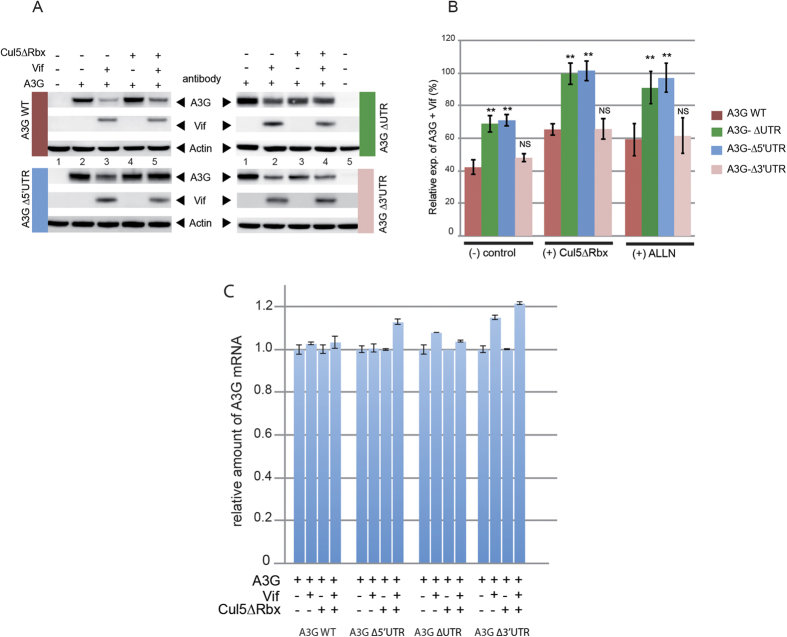
Vif inhibits A3G translation in a 5′UTR dependent manner. (**A**) HEK 293T cells were transfected with plasmids expressing A3G from different mRNA constructs in the presence or absence of Vif expression and in the presence or absence of proteasome inhibitors (Cul5∆Rbx or ALLN). (**B**) Quantification of the relative expression of A3G. (**C**) Total RNA was extracted from wild-type and mutant A3G transfected HEK 293T cells and A3G RT-qPCR was performed to study the relative expression of A3G constructs. Standard deviations are representative of at least three independent experiments. P-values are indicated as follows: *<0.05, **<0.01, NS: non significant. Blots have been cropped and full-length blots are presented in Supplementary Figure S2. All samples derive from the same experiment and blots were processed in parallel.

In a work using biochemical and *in vitro*-coupled transcription/translation assays^40^, we previously showed that Vif was able to bind the UTRs of A3G mRNA with high affinity and pointed out the importance of A3G 5′-UTR in its translational inhibition by Vif. Here, our first goal was thus to test whether a similar inhibition could be observed in human cell lines. First, we examined the level of A3G expressed in transfected HEK 293T cells from full-length A3G mRNA (containing 5′ and 3′-UTRs) or from mutant mRNAs deleted of their 5′, 3′ or 5′ plus 3′-UTRs (Figs 1 and 2), in presence or in absence of Vif expression. To discriminate the effects of Vif on A3G translation from its well documented effect on A3G degradation, we performed all experiments in presence or absence of a dominant negative mutant of Cul5 (Cul5∆Rbx), which has previously been shown to specifically inhibit A3G degradation through the proteasome pathway^25^, or by using ALLN, a potent chemical proteasome inhibitor also used to study A3G proteasomal degradation^36^,^37^. Moreover, to avoid saturating our system by overexpressed concentrations of A3G, we always transfected a pCMV-A3G/pcDNA-Vif (or pNL4.3) ratio of 1/20 (50 ng pCMV-A3G)^42^.

**Figure 3:**
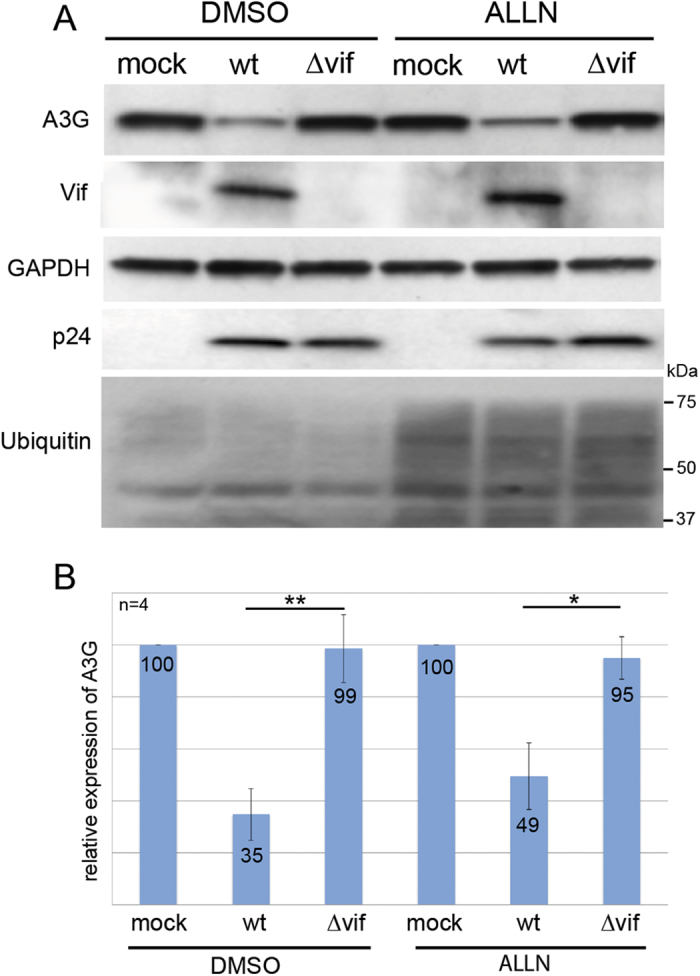
Vif inhibits A3G translation in HIV-1 chronically infected H9 cells. (**A**) Wild-type and chronically infected H9 cells (HXB2 wild-type and HXB2∆vif) were cultured in absence (DMSO) or presence of proteasome inhibitor (ALLN) for 18 h and analyzed by western-blot with specific antibodies against A3G, Vif, GAPDH, p24 and ubiquitin (see Material and Methods). (**B**) Quantification of the relative expression of A3G. Standard deviations are representative of at least four independent experiments. P-values are indicated as follows: *<0.05, **<0.01. Blots have been cropped and full-length blots are presented in Supplementary Figure S3. All samples derive from the same experiment and blots were processed in parallel.

Immunoblotting of normalized cell lysates with an A3G-specific antibody revealed that the expression of A3G from wild-type mRNA was reduced by 60% in the presence of Vif (Fig. 2A, left upper panel and Fig. 2B, control histogram for quantitation). When we analyzed the UTR-truncated versions of A3G mRNA, we observed a similar decrease when the 3′UTR of A3G mRNA was deleted (50–60% decrease) (Fig. 2A, right lower panels and Fig. 2B, control histogram). Remarkably, this decrease was twofold less pronounced (around 30%) when the 5′-or both UTRs were deleted (Fig. 2A and B, control histogram). Interestingly, when A3G degradation by the proteasome was blocked by using either a dominant negative mutant of Cul5 (Cul5∆Rbx) (Fig. 2A and B, central histogram) or a chemical inhibitor (ALLN) (Fig. 2B, right histogram), we still observed a significant decrease of A3G (30–40%) expressed from wild-type or ∆3′UTR mRNAs, while no decrease of A3G expressed from ∆5′UTR and ∆UTRs mRNAs was observed in the presence of Vif. Consistent with previous studies^36^,^37^,^43^, Vif did not affect A3G mRNA levels, which remained constant in all conditions and for all A3G mRNA constructs (Fig. 2C), indicating that variations of the A3G protein level were not due to differential transcription of the vectors or to mRNA degradation. Secondly, in order to test the translational effect of Vif in a more physiological context, we directly analyzed the level of A3G expressed in HIV-1 chronically infected H9 cells^44^ in presence or in absence of ALLN proteasome inhibitor (Fig. 3). These cells expressed normal amounts of viral proteins (Vif and capsid p24) and a higher amount of total ubiquinated proteins could be observed when the proteasome was inhibited (Fig. 3A). As expected from a Vif-induced degradation of A3G, HXB2 infected H9 cells (Vif+) expressed a lower level of A3G protein (Fig. 3A), with a decrease of about 70% (Fig. 3B, left histogram). Importantly, inhibition of the proteasome with ALLN did not completely restore the expression of A3G, as a 49% inhibition was still observed (Fig. 3A and B), suggesting that Vif inhibits translation of A3G. We did not observe any effect on the degradation of A3G protein, or on the translation of its mRNA, in absence of Vif in this context (HXB2∆vif chronically infected cells) (Fig. 3). Altogether, these results demonstrate that Vif inhibits A3G translation in H9 infected and HEK 293T transfected cells in a 5′UTR dependent manner and that this translational inhibition could be a quantitatively important mechanism compared to proteasomal degradation. In other words, Vif-induced proteasomal degradation and translational inhibition by Vif are both efficient processes.

### The 5′UTR of A3G mRNA cannot be replaced by heterologous 5′UTRs

**Figure 4:**
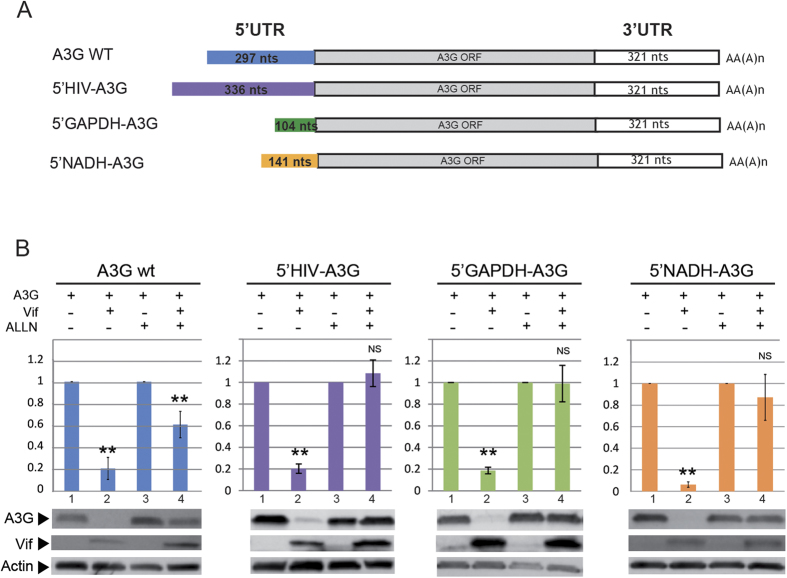
Heterologous 5′UTRs do not allow inhibition of A3G translation by Vif. (**A**) Schematic representation of A3G expression constructs containing wild-type or heterologous (HIV-1, GAPDH and NADH) 5′UTR. (**B**) HEK 293T cells were transfected with these 5′UTR vectors in the presence or absence of Vif expression and in the presence or absence of proteasome inhibitors (ALLN). Quantification of the relative expression of A3G is represented by histograms. Standard deviations are representative of at least three independent experiments. P-values are indicated as follows: *<0.05, **<0.01, NS: non significant. Blots have been cropped and full-length blots are presented in Supplementary Figure S4. All samples derive from the same experiment and blots were processed in parallel.

The results presented above suggest that reduced translation of A3G in presence of Vif could be attributed to the 5′UTR of its mRNA. To further confirm these observations, we compared the Vif-induced translational inhibition of A3G mRNA containing its native 5′UTR to the one of A3G mRNAs bearing the 5′UTR from different cellular and viral transcripts, such as GAPDH, NADH and HIV-1 (Fig. 4A). Interestingly, while we showed a clear degradation of A3G protein for all tested constructs in absence ALLN (Fig. 4B, compare bar 2 to bar 1), no significant reduction of A3G expression (degradation and/or translation) could be observed when the proteasome was inhibited, except for the native A3G mRNA (Fig. 4B, compare bar 4 to bar 3). These results confirm that the 5′UTR of A3G mRNA is a major element in the process and that the translational inhibition mediated by Vif strictly requires this 5′UTR.

### Vif requires stem-loops 2 and 3 of A3G mRNA to inhibit translation

**Figure 5:**
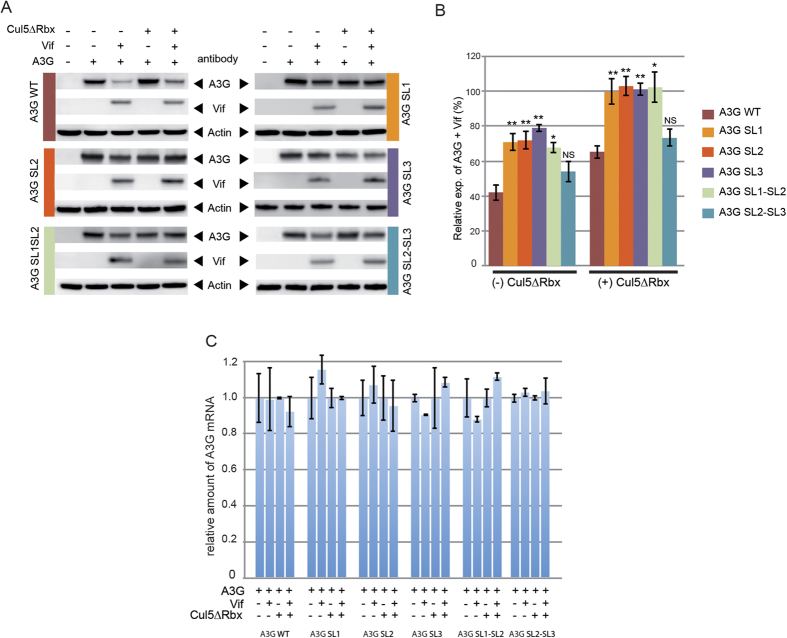
Vif requires SL2 and SL3 to impair A3G translation. HEK 293T cells were transfected with wild-type or mutated A3G mRNA constructs and co-transfected in the presence or absence of Vif expression and in the presence or absence of proteasome inhibitors (Cul5∆Rbx). (**A**) Proteins were separated by SDS-PAGE and analyzed by immunoblotting. (**B**) Quantification of the relative expression of A3G. (**C**) Total RNA was extracted from wild-type and mutant A3G transfected HEK 293T cells and A3G RT-qPCR was performed to study the relative expression of A3G constructs. Standard deviations are representative of at least three independent experiments. P-values are indicated as follows: *<0.05, **<0.01, NS: non significant. Blots have been cropped and full-length blots are presented in Supplementary Figure S5. All samples derive from the same experiment and blots were processed in parallel.

The 5′UTR of A3G mRNA contains three independent stem-loop (SL) motifs (Fig. 1), and Vif binds to a few high-affinity binding sites in this region^40^. In order to identify the domains in the 5′UTR that are required for A3G translational inhibition by Vif, we designed A3G mRNA constructs containing each of the individual SL motifs (A3G SL1, containing only SL1, A3G SL2 and A3G SL3) or two consecutive SL motifs (A3G SL1-SL2 and A3G SL2-SL3) (Fig. 1). In absence of proteasome inhibition, Vif more strongly affected expression of A3G from wild-type and A3G SL2-SL3 mRNAs than from the other mRNA constructs (50–60% *versus* 20–30%) (Fig. 5A and B, left histogram). When proteasomal degradation was inhibited, A3G levels were reduced in the presence of Vif only when it was expressed from wild-type or A3G SL2-SL3 mRNAs (by 30–40%) (Fig. 5B). Again, changes in the A3G protein levels were not due to variations in the A3G mRNA levels (Fig. 5C). Thus, our data indicated that both the SL2 and SL3 motifs within the 5′UTR of A3G mRNA are required to allow A3G translational inhibition by Vif.

### Residue K26 of Vif is required for A3G degradation by the proteasome but not for A3G translation inhibition

**Figure 6:**
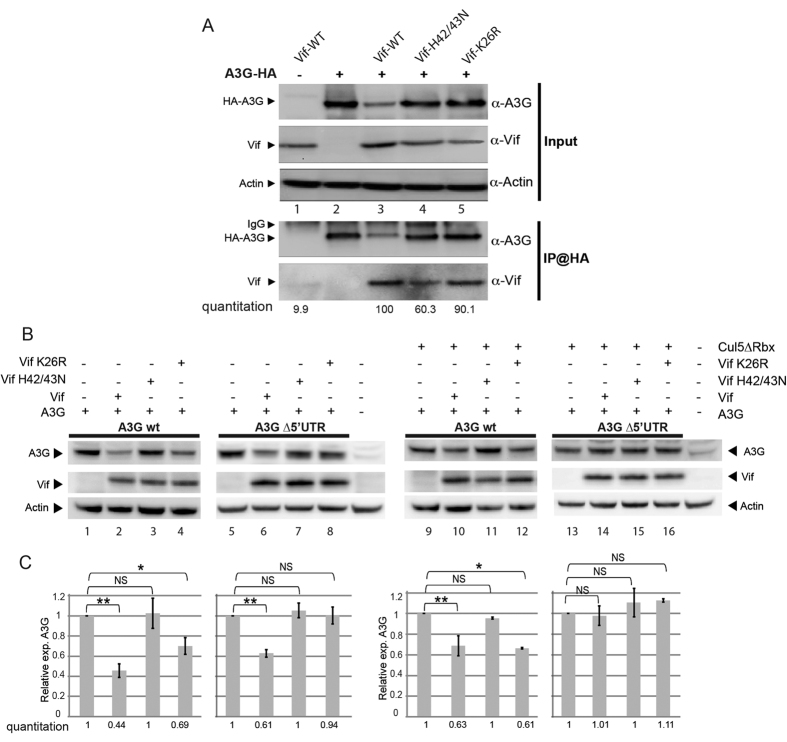
Vif K26 residue is required for the translational inhibition of A3G. (**A**) HEK 293T cells were transfected with plasmids expressing A3G-HA in the presence of wild-type of mutant (H42/43N and K26R) Vif expression vectors. The input fractions were revealed by anti-Vif, anti-HA, and anti-Actin antibodies. Immuno-precipitation assays were performed using an anti-HA antibody directed against the A3G-HA protein. A negative control without A3G-HA (lane 1), and a specificity control without Vif (lane 2) were also included. The fraction of Vif proteins interacting with A3G-HA is indicated. (**B**) HEK 293T cells were co-transfected with wild-type or ∆5′UTR A3G mRNA expression vectors and with wild-type, K26R or H42/43N Vif expressing vectors. Proteins were separated by SDS-PAGE and analyzed by immunoblotting. (**C**) Quantification of relative A3G expression. Standard deviations are representative of at least three independent experiments. P-values for the different assays are indicated as follows: *<0.05, **<0.01, NS: non significant. Blots have been cropped and full-length blots are presented in Supplementary Figure S6. All samples derive from the same experiment and blots were processed in parallel.

Next, we tested the impact of two mutations in Vif that have been shown to impair A3G degradation on the inhibition of A3G translation by Vif (Fig. 6). According to the literature, Vif mutant K26R is still able to interact with A3G^45^,^46^,^47^, while mutant H42/43N is defective for A3G binding, while retaining binding to the CRL5 complex^48^. First, we tested the interaction of these two Vif mutants with HA-tagged A3G by co-immunoprecipitation (Fig. 6A), and we observed that Vif K26R retained its binding capacities with A3G while the binding of Vif H42/43N was decreased ~twofold. Next, we observed that when proteasomal degradation of A3G was not inhibited, expression of A3G from wild-type mRNA was partially reduced (30–40%) by Vif K26R, in comparison to wild-type Vif (60% reduction) (Fig. 6B, compare lanes 2 & 4, and Fig. 6C), whereas Vif H42/43N did not reduce the A3G protein level (Fig. 6B & C, lane 3). When A3G was expressed from the ∆5′UTR mRNA construct, which is not sensitive to translational inhibition by Vif, the two Vif mutants did not induce any decrease in A3G protein level (Fig. 6B, lanes 7 & 8), confirming that these mutants are unable to induce A3G degradation by the proteasome. Importantly, when proteasomal degradation was blocked, wild-type and K26R Vif significantly and similarly inhibited translation of A3G (30–40%) when expressed from wild-type mRNA (Fig. 6B & C, lanes 10 & 12), whereas Vif H42/43N did not reduce A3G levels (Fig. 6B, lane 11). When A3G was expressed from the ∆5′UTR mRNA under the same conditions, A3G protein level was not affected by the wild-type or mutant Vif proteins (Fig. 6B & C, lanes 13 to 16). Altogether, these results demonstrate that Vif K26R, while defective in A3G degradation through the proteasome pathway, is still fully able to decrease A3G expression through inhibition of A3G mRNA translation, thus demonstrating that these two processes are independent. By contrast mutation H42/43N inhibits both pathways.

### The Vif-induced translational inhibition reduces packaging of A3G

**Figure 7:**
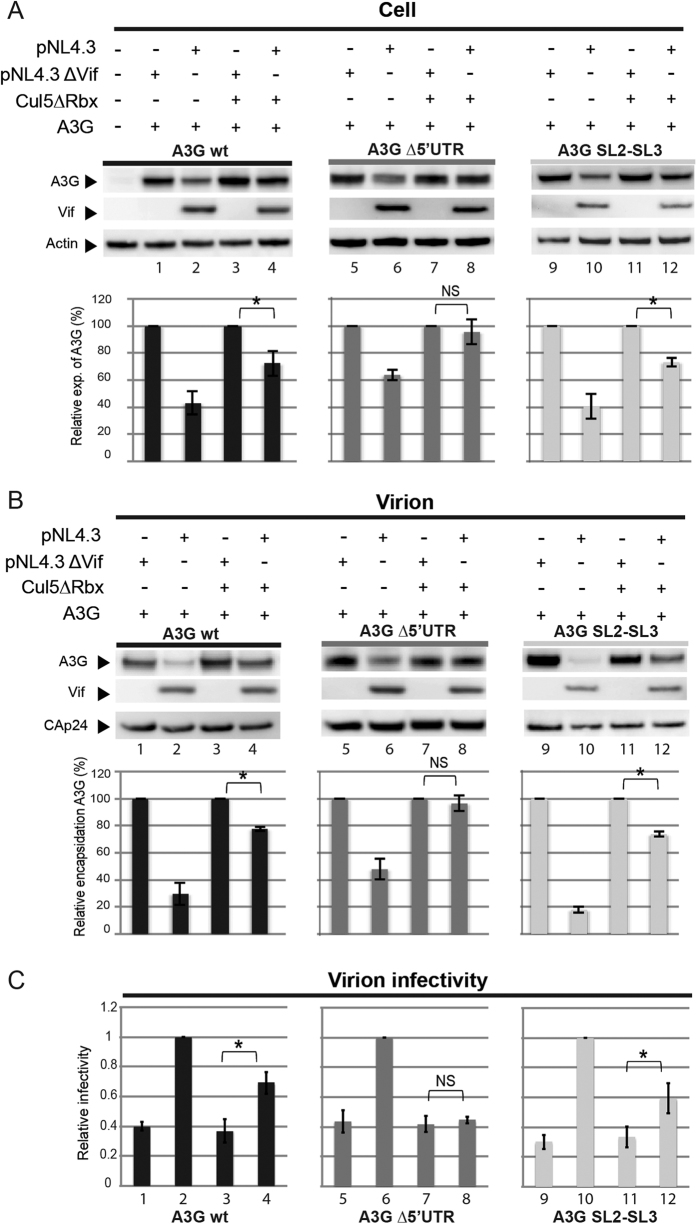
Effect of the inhibition of A3G translation by Vif on A3G packaging and viral infectivity. HEK 293T cells were co-transfected with HIV-1 pNL4.3 or pNL4.3Δvif and wild-type or mutant A3G mRNAs +/− proteasome inhibitor Cul5∆Rbx. Proteins from cell lysates (**A**) and virions (**B**) were separated by SDS-PAGE and analyzed by immunoblotting. (**C**) Viral particles produced in HEK 293T cells were used in viral infectivity assay using TZM-bl indicator cells. Luciferase induction was detected 48 h post-infection. Standard deviations are representative of at least three independent experiments. P-values for the different assays are indicated as follows: *<0.05, **<0.01, NS: non significant. Blots have been cropped and full-length blots are presented in Supplementary Figure S7. All samples derive from the same experiment and blots were processed in parallel.

To investigate the impact of the inhibition of the translation of A3G by Vif on its packaging into viral particles, we co-transfected wild-type (pNL4.3) or Vif deleted (pNL4.3∆vif) molecular clones of HIV-1 together with full-length, ∆5′UTR or SL2-SL3 A3G expression vectors (Fig. 7). Note that amongst these constructs, only A3G ∆5′UTR is not sensitive to the translational regulation by Vif (Fig. 7A, middle panel). These experiments were performed in presence of the dominant negative mutant of Cul5 (Cul5∆Rbx). Cell lysates (Fig. 7A) and concentrated virus fractions (Fig. 7B) were analyzed by immunoblotting with specific Vif, A3G and CAp24 antibodies (see Material and Methods). Consistent with results in Figs 2A and 5A, analysis of the A3G expression levels showed that inhibition of A3G translation and degradation by the proteasome were equally potent in reducing the intracellular A3G content when Vif was expressed from a proviral molecular clone, as each mechanism contributed to reduce the A3G level by ~35% (Fig. 7A, compare lanes 2 & 4 for wild-type A3G and lanes 10 & 12 for A3G SL2-SL3).

Next, we analyzed the packaging of A3G proteins into viral particles (Fig. 7B). We observed a direct correlation between the A3G expression level in cells (Fig. 7A) and its incorporation into viral particles (Fig. 7B). In all cases, reduction in the intracellular A3G levels resulted in decreased incorporation of this restriction factor into the viral particles, independently of the mechanism (inhibition of translation or proteasomal degradation) reducing A3G expression. Note that when the proteasomal degradation and translational repression were both inhibited by expressing Cul5∆Rbx and deleting the 5′UTR of A3G mRNA, respectively, the intravirion A3G level was not modified by Vif (Fig. 7B, lanes 7 & 8). Thus, these two mechanisms significantly contribute to exclude A3G from viral particles. In agreement with this conclusion, the amount of Vif protein encapsidated into viral particles was constant under all conditions studied, indicating that a direct competition between Vif and A3G for packaging is unlikely.

### The inhibition of A3G translation by Vif restores viral infectivity

To determine whether the repression of A3G translation by Vif impacts HIV-1 infectivity, wild-type (pNL4.3) or Vif-defective (pNL4.3∆vif) virus stocks were produced in HEK 293T cells co-transfected with wild-type, ∆5′UTR or SL2-SL3 A3G mRNA constructs in presence or absence of Cul5∆Rbx (Fig. 7C). The viral infectivity was determined by infecting TZM-Bl indicator HeLa cells after normalization of virus stocks^49^. As expected, viral infectivity was strongly decreased in the absence of Vif (Fig. 7C, lanes 1, 5 & 9 in the absence of Cul5∆Rbx, and 3, 7 & 11 in the presence of Cul5∆Rbx). Interestingly, we observed a partial but significant restoration of viral infectivity in presence of Vif even when A3G degradation was inhibited, when A3G was expressed from wild-type and SL2-SL3 mRNA, i.e. from mRNAs that are sensitive to translation inhibition by Vif (Fig. 7C, lanes 4 & 12). However, under these conditions, Vif was not able to restore the HIV-1 infectivity when A3G was translated from an mRNA deleted from its 5′UTR (Fig. 7C, compare lanes 7 & 8). These results indicate that the inhibition of A3G translation by Vif is sufficient to partially restore HIV-1 infectivity.

### Most HIV-1 Vif alleles induce a translational down-regulation of A3G

**Figure 8:**
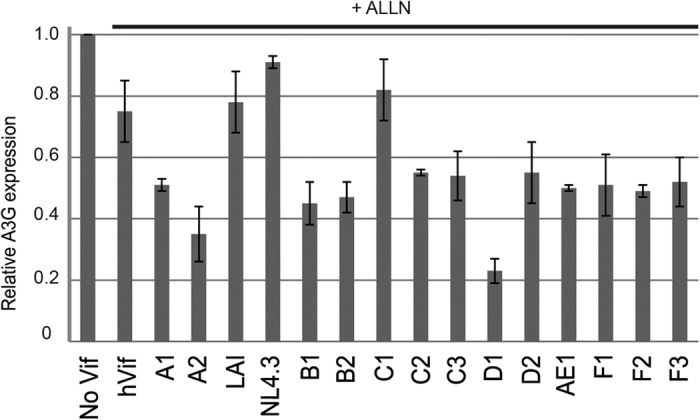
Translational repression of A3G by different Vif alleles. HEK 293T cells were transfected with plasmids expressing wild-type A3G mRNA in the presence of Vif alleles and proteasome inhibition (ALLN), and the relative A3G expression was analyzed by western blot and quantify using Image J (1.46r). Standard deviations are representative of at least three independent experiments.

Although Vif is expressed by all HIV-1 strains, its sequence varies considerably amongst HIV-1 isolates (Los Alamos HIV sequence database, http://www.lanl.gov/). To test whether the Vif-induced translational inhibition of A3G is a general property of HIV-1 Vif, we analyzed by western blot the relative expression of A3G after co-transfection of HEK 293T cells with different Vif alleles^50^ and wild-type A3G, in presence or absence of proteasome inhibitor. Expression of A3G alone (no Vif) was set to 100%, and we chose hVif as a reference for comparison to other Vif proteins as it was used all along in our study (Fig. 8). As previously observed (Fig. 2B), the expression of A3G from wild-type mRNA in presence of ALLN was reduced by 30–40% in presence of hVif. Three Vif variants (LAI, NL4.3, and C1) displayed lower inhibition of A3G translation (10–30% inhibition), whereas eleven (A1, A2, B1, B2, C2, C3, D2, AE1, F1–3) produced a stronger inhibition (40–80%) going to up to 80% in the case of the D1 Vif allele. The fact that the level of A3G inhibition mediated by Vif NL4.3 was a bit lower than the one observed in Fig. 7A (with pNL4.3 molecular clone) probably originated from the expression vectors used (see Material and Methods). Altogether, these results demonstrate that the translational inhibition of A3G is a common property of Vif, but the degree of regulation differs from one HIV-1 isolate to the other.

## Discussion

The HIV-1 Vif protein has been shown to be necessary for efficient viral infection in non-permissive cells by antagonizing the antiviral activity of A3G. While the molecular mechanisms by which Vif induces degradation of A3G by the proteasome have been extensively studied since the discovery of A3G^43^, little is known about the regulation of A3G translation by Vif. Using *in vitro* coupled transcription/translation, we previously showed that inhibition of A3G translation by Vif requires the 5′UTR of the A3G mRNA^40^, but the relative importance of the inhibition of A3G translation and its degradation, both induced by Vif, could not be determined in this study. Here, compared to studies by other groups, we were able to address the translational effect of Vif on A3G by using expression vectors containing the authentic 5′UTR of A3G mRNA (and not heterologous sequences brought by the plasmid). We thus observed that A3G expression is diminished by Vif in HEK 293T transfected and in H9 infected cells, even when its degradation is blocked, provided this restriction factor is expressed from an mRNA containing the complete 5′UTR (Fig. 2) or at least structural domains SL2 and SL3 (Fig. 5). Thus, this phenomenon most likely corresponds to the inhibition of translation we previously observed *in vitro*^40^.

Interestingly, *in vitro* foot-printing experiments^40^ identified several Vif-binding sites in the SL3 motif, suggesting that Vif may slow-down the ribosome scanning process on the 5′UTR of A3G mRNA, thus reducing A3G translation. However, SL2 was required for inhibition of A3G translation by Vif (Fig. 5), even though no Vif binding site was observed in this region^40^, raising the possibility that the mechanism is more complex. In any case, specific structural elements in the 5′UTR of A3G mRNA are likely required for an optimal down-regulation of A3G translation since its 5′UTR could not be substituted for heterologous cellular or viral 5′UTRs varying in size and complexity (Fig. 4).

Although specific amino acids in Vif have been shown to be necessary for A3G binding and proteasomal degradation^27^, so far none have been identified as being required for inhibition of A3G translational translation. Importantly, we observed that Vif mutant K26R, in the N-terminus of Vif, while unable to induce A3G degradation by the proteasome was fully able to inhibit A3G translation, demonstrating that these two pathways are independent (Fig. 6). Notably, Vif mutant H42/43N, which unlike mutant K26R, displayed decreased interaction with A3G, was unable to inhibit translation, raising the possibility that Vif/A3G interaction might be required for translational regulation.

By using A3G mRNA constructs that allowed or prevented inhibition of A3G translation by Vif, together with inhibitors of A3G proteasomal degradation, we could estimate the relative contributions of these two processes to the overall decrease of A3G intracellular and intravirion concentrations induced by Vif (Figs 2, 5 and 8). Importantly, we observed that these two processes contribute to the decrease of A3G levels in cells and virions, suggesting that the translational inhibition could be a quantitatively important mechanism compared to proteasomal degradation. Thus, the inhibition of A3G translation by Vif is an important process, which was not detected in most previous studies due to the use of A3G expression vectors lacking the authentic 5′UTR, and thus preventing this translational control of A3G by Vif. Moreover, this property is shared by a large number of Vif alleles, with differential translational activity (Fig. 8), suggesting that specific sequences/domains of Vif are required to down-regulate A3G translation.

In addition to inducing degradation of A3G and preventing its translation, it has been suggested that Vif could reduce A3G incorporation into viral particles by directly interfering with its packaging possibly by competing for a common RNA motif required for packaging of these two proteins^51^,^52^,^53^,^54^. However, our results show that when degradation of A3G by the proteasome and inhibition of A3G translation are both blocked, the intracellular and intravirion concentrations of A3G are the same as in the absence of Vif (Fig. 7A and B), indicating that these two mechanisms are the only significant pathways by which Vif reduces A3G incorporation into virions. At the opposite, basal levels of A3G were incorporated into viral particles even when both pathways were active (Fig. 7B).

Finally, we showed that inhibition of A3G translation by Vif is able to partially restore infectivity of the HIV-1 particles in the absence of degradation of this restriction factor (Fig. 7C). This is the first demonstration of the functional role of the translational control of A3G by Vif in the context of viral infection. The results of our experiments using reporter cells are corroborated by previous studies^46^,^48^,^55^ showing that an HIV-1 mutant bearing mutation K26R in the *vif* gene replicated in restrictive cells, while a virus with the H42/43N mutations did not. Combined with our finding that the first mutation did abolish A3G translational control by Vif, while the second did not (Fig. 6), these results further prove that inhibition of A3G translation by Vif is sufficient to partially restore HIV-1 infectivity. Thus, our findings demonstrated that the translational inhibition of A3G by Vif could be considered as a third layer of *A3G* gene regulation in addition to its protein degradation and transcriptional down-regulation^28^. This translational control is corroborated by the fact that this property is shared by almost all Vif proteins and opens attractive perspectives for the development of new drugs disrupting the translational control of A3G by Vif. Moreover, considering that the 5′UTR of A3G and A3F mRNAs is highly conserved, it is likely that Vif is also able to inhibit A3F translation.

## Methods

### Plasmids

Plasmids pCMV-hA3G, pCMV-hA3GΔUTR, pCMV-hA3GΔ5′UTR and pCMV-hA3GΔ3′UTR have been previously described^40^. Contrary to expression vectors used in the literature (in which the A3G open reading frame was fused to the heterologous 5′ and 3′ UTRs of the expression plasmid), our wild-type construct (pMCV-hA3G) expresses the full-length cellular A3G mRNA containing authentic 5′ and 3′-UTRs, in order to recapitulate events occurring at the translational level^38^,^39^. pCMV-hA3G SL1 (deletion of nucleotides 112 to 297, ∆112–297), SL2 (∆1–128 and ∆199–297), SL3 (∆1–207), SL1SL2 (∆199–297) and SL2SL3 (∆1–127) were generated by Quick-Change Site-directed Mutagenesis (Agilent Technologies) based on the secondary structure model of the 5′-UTR of hA3G mRNA^40^ and deletions were confirmed by DNA sequencing (GATC Biotech, Germany). Vif was expressed from plasmid pcDNA hVif encoding codon-optimized NL4.3 Vif^56^ which is available through the NIH AIDS Research and Reference Reagent Program (catalog #10077). The different alleles of Vif were obtained from Dr. M. Ooms (Department of Microbiology, Mount Sinai School of Medicine, New York, USA)^50^ and expressed Vif from a hybrid expression vector (pCRV1) that is derived from pCR3.1 (life technologies). Mutations K26R and H42/H43-N were generated in the pcDNAhVif plasmid by Quick-Change Site-directed Mutagenesis (Agilent Technologies). The infectious molecular clone pNL4–3 has been previously described^57^ and is also available through the NIH AIDS Research and Reference Reagent Program (catalog #114). The vif-defective pNL4-3 variant, pNL4-3Δvif, carrying a 178-bp out-of-frame deletion in the *vif* gene, has been previously reported^58^. A plasmid expressing a dominant negative mutant of Cul5, pCul5ΔRbx, was used to block A3G degradation by the proteasome^25^. pNL4.3, pNL4.3Δvif, pNL4.3Δenv and pNL4.3ΔenvΔvif were generously provided by Dr. Klaus Strebel (National Institutes of Health, Bethesda, MD). The pCMV A3G-derived constructs containing the heterologous 5′UTR from NADH, GAPDH and HIV-1 (NL4.3) were obtained by ligating the EcoRI-PstI PCR amplified products from NADH (141 nts) and GAPDH (104 nts) cDNAs (kindly provided by Dr T. Ohlmann, INSERM U758 Lyon, France^59^), and HIV-1 pNL4.3 (336 nts), respectively, into the double digested pCMV A3G expression vector. All constructs were confirmed by DNA sequencing (GATC Biotech, Germany).

### Cell Culture, transfection and infection

HEK 293T and TZM-bl cells cells were maintained in Dulbecco’s modified Eagle’s medium (DMEM, Life Technologies) supplemented with 10% fetal bovine serum (FBS, EUROBIO) with antibiotics (PAA) and passaged upon confluence. Transfections of HEK 293T cells were carried out using the X-tremeGENE 9 DNA Transfection Reagent (Roche) as recommended by the manufacturer. Briefly, cells were seeded at 70% confluence in a 6-well plate and co-transfected with 50 ng of pCMV-hA3G constructs, 1 μg of pcDNA hVif, hVif mutants or the different Vif alleles with or without 0.5 μg of pCul5ΔRbx. In parallel experiments, cells were exposed to the chemical proteasome inhibitor ALLN (25 μM) or DMSO (control) for 14 h. For HIV-1 chronically infected H9 cells analysis, three T-cell lines were used: wild-type H9 (mock); H9 HXB2∆env and H9 HXB2∆env∆vif containing an integrated HIV-HXB2 provirus deleted for *Env* and for both *Env* and *Vif* genes, respectively. These constructions carry the neomycin phospho-transferase gene (*neo*^*R*^) cloned into the *nef* region of the genome. Chronically infected cells were grown in RPMI medium supplemented with 10% FBS and G418 (1 mg/ml)^44^. As for HEK 293T cells, H9 cells were exposed to ALLN (25 μM) or DMSO for 14 h. For infectivity assays and study of A3G incorporation into HIV-1 particles, HEK 293T cells were co-transfected with 50 ng of pCMV-hA3G mutants, 0.5 μg of Cul5ΔRbx and 1 μg of HIV-1 pNL4.3 (pNL4.3 wt, pNL4.3Δvif). For infectivity assays, TZM-bl indicator cells were challenged with reverse transcriptase normalized virions and the induction of luciferase was detected 48 h post-infection^60^.

### Immuno-precipitation assays

HEK 293T cells were co-transfected with pA3G-HA and plasmids expressing wild type or mutant Vif, Twenty-four h post-transfection, cells were washed in PBS (140 mM NaCl, 8 mM NaH_2_PO_4_, 2 mM Na_2_HPO_4_) and lysed in RIPA 1X (PBS, 1% NP40, 0.5% sodium deoxycholate, 0.05% SDS) supplemented with protease inhibitors (cOmplete EDTA Free cocktail, Roche). After centrifugation, an aliquot fraction (50 μl) was used for determination of the protein expression level, and the remaining was incubated 2 h at 4 °C with 1 μg of HA antibody (Santa Cruz, California, USA) on a rotating wheel. After equilibration, protein A Dynabeads (Life Technologies) were added and incubated for 1.5 h at 4 °C. Beads were washed 5 times with cold RIPA, and eluted in glycine pH 2.8, NuPAGE LDS sample buffer (Life Technologies). After 10 min at 70 °C, supernatant was loaded on NuPAGE gel (Life Technologies) and analyzed by western blot.

### Immunoblotting

Twenty-four hours post-transfection, cells were harvested in RIPA supplemented with protease inhibitors. Virions from transfected 293T cells were concentrated by ultracentrifugation at 100,000 g for 2 h at 4 °C through a 20% sucrose cushion and harvested in RIPA. Cell and virions lysates were adjusted to equivalent protein concentration (determined using Bradford reagent (BIO-RAD), fractionated on NuPAGE® Novex® 4–12% Bis-Tris gels (Life Technologies) and transferred to a 22 μm PVDF membranes using the Trans-Blot® Turbo™ Transfer System (BIO-RAD). Western blot membranes were cut to minimize antibody usage (Supplementary Figure 1) and probed with appropriate primary antibodies. Polyclonal anti-hA3G (#9968) and monoclonal anti-HIV-1 Vif (#319) antibodies were obtained through the NIH AIDS Research and Reference Reagent Program. Monoclonal anti-β-actin antibody was purchased from SIGMA (#A5316). An HIV-positive patient serum was used for the identification of HIV-1 p24 protein. The PVDF membranes were then incubated with horseradish peroxidase-conjugated secondary antibodies (BIO-RAD), and the proteins were visualized by enhanced chemiluminescence (ECL) using the ECL Prime Western blotting detection reagent (GE Healthcares). Bands were quantified using Image J (1.46r) by analyzing pixel density. Student’s T-test was used to determine statistical significance.

For H9 T-cells, 5.10^6^ cells (treated or not with ALLN/DMSO) were harvested by centrifugation and lysed for 10 min at 4 °C in RIPA 1X supplemented with protease inhibitors. Lysates were cleared by centrifugation for 30 min at 14,000 g and protein concentration was determined using a Bradford assay in order to load the equivalent of 150 μg of total proteins on a NuPAGE® Novex® 4–12% Bis-Tris gels (Life Technologies). Western blot was then performed as above using antibodies directed against A3G (NIH#9968), Vif (NIH#319), GAPDH (ABD Serotec-Bio-Rad); p24 (HIV-positive patient serum) and Ubiquitin (Santa Cruz, California, USA). Appropriate HRP-conjugated secondary antibodies were used and revealed by chemiluminescence, and bands were quantified as above.

### RNA isolation, cDNA synthesis and real-time qPCR

Twenty-four h post-transfection, total RNA was isolated from 293T cells using TRI Reagent (SIGMA). After RNase-free DNAse treatment (Roche), total RNA was isolated by phenol/chloroform extraction followed by ethanol precipitation. Total RNA (1 μg) was then reverse-transcribed using the iScript^TM^ Reverse Transcription Supermix (BIO-RAD) as recommended by the manufacturer. Subsequent qPCR analysis was performed using the KAPA SYBR® FAST qPCR Master Mix (KAPA BIOSYSTEMS) and was monitored on a CFX Real Time System (BIO-RAD). Gene-specific primers were: A3G forward primer, 5′-TCCACCCACATTCACTTTCA-3′, and reverse primer 5′-TTCCAAAAGGGAATCACGTC-3′; β-actin forward primer, 5′-GGACTTCGAGCAAGAGATGG-3′, and reverse primer 5′-AGCACTGTGTTGGCGTACAG-3′. The A3G mRNA levels were normalized to those of β-actin mRNA and relative quantification was determined using the standard curve based method.

## Additional Information

**How to cite this article**: Guerrero, S. *et al*. Translational regulation of APOBEC3G mRNA by Vif requires its 5′UTR and contributes to restoring HIV-1 infectivity. *Sci. Rep.* **6**, 39507; doi: 10.1038/srep39507 (2016).

**Publisher's note:** Springer Nature remains neutral with regard to jurisdictional claims in published maps and institutional affiliations.

## Supplementary Material

Supplementary Information
