# Supplementary material for: Translational regulation of APOBEC3G mRNA by Vif requires its 5′UTR and contributes to restoring HIV-1 infectivity
Source: Sci Rep. 2016 Dec 20;6:39507. doi: 10.1038/srep39507 (PMC5171582; doi:10.1038/srep39507)

Translational regulation of APOBEC3G mRNA by Vif requires its 5'UTR and contributes to restoring HIV-1 infectivity

Santiago Guerrero[1†], Camille Libre[1], Julien Batisse[1#], Gaëlle Mercenne[1‡], Delphine Richer[1], Géraldine Laumond[2], Thomas Decoville[2], Christiane Moog[2], Roland Marquet[1] & Jean-Christophe Paillart[1*]

[1] Université de Strasbourg, CNRS, Architecture et Réactivité de l'ARN, UPR 9002, IBMC-15 rue René Descartes, F-67000, Strasbourg, France

[2] Université de Strasbourg, INSERM, UMR 1109, Laboratoire d'ImmunoRhumatologie Moléculaire, Fédération de Médecine Translationnelle de Strasbourg (FMTS), Institut de Virologie, 3 rue Koeberlé, F-67000 Strasbourg, France

# Corresponding author: Dr. Jean-Christophe Paillart

e-mail: jc.paillart@ibmc-cnrs.unistra.fr

Phone: + 33 3 88 41 70 35 ; Fax: + 33 3 88 60 22 18

Present addresses: [†]Gene Regulation, Stem Cells and Cancer Programme, Centre for Genomic Regulation (CRG), The Barcelona Institute for Science and Technology, Barcelona, Spain, Universitat Pompeu Fabra (UPF), Barcelona, Spain. [‡]Recursion Pharmaceutical, LLC, 283 S. Colorow Drive, Salt Lake City, UT 84108-5650, USA. [#] IGBMC, Université de Strasbourg, CNRS, INSERM, 1 rue Laurent Fries, 67404 Illkirch, France

Supplementary Figure S1. Example of western blot membrane that was cut as indicated to minimize antibody usage.

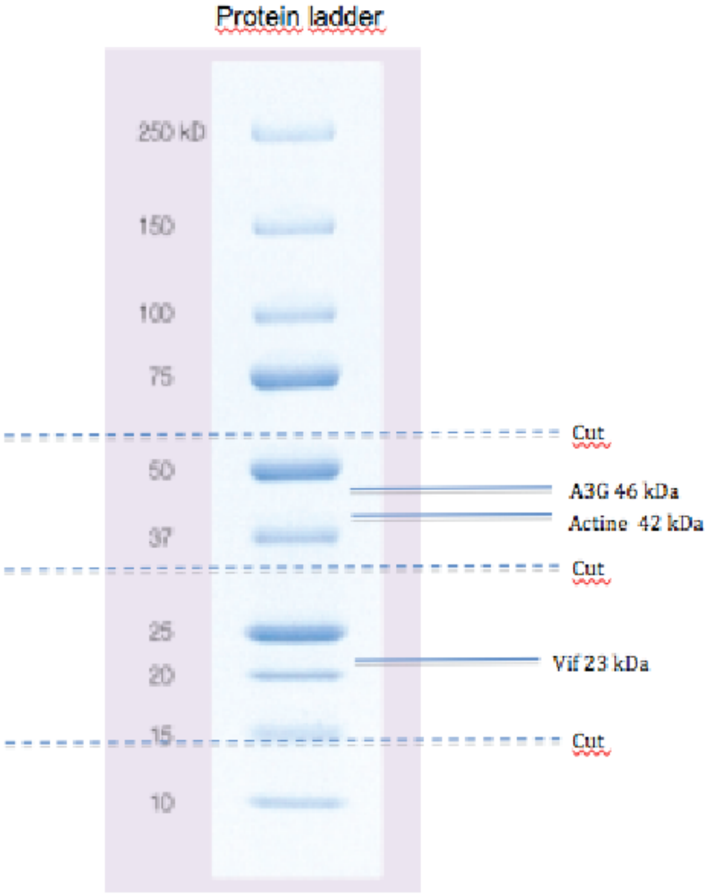

Supplementary Figure S2. Vif inhibits A3G translation in a 5'UTR dependent manner. Full-length blots of Figure 2 showing A3G, Vif, with the actin control that was run on the same gel. Arrows point to the indicated proteins. Parts that have been cropped are boxed.

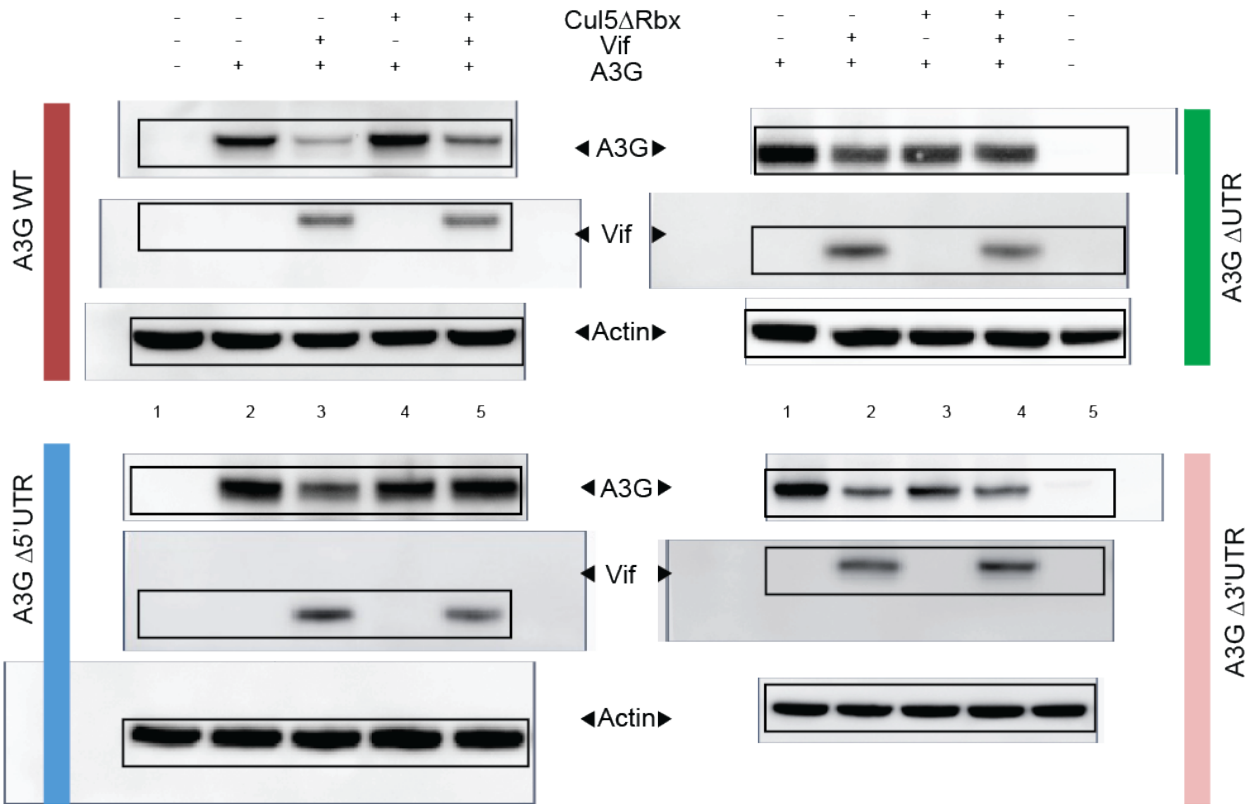

Supplementary Figure S3: Vif inhibits A3G translation in HIV-1 chronically infected H9 cells. Full-length blots of Figure 3, showing A3G, Vif, p24, Ubiquitin with the GAPDH control that was run on the same gel. Arrows point to the indicated proteins. Parts that have been cropped are boxed.

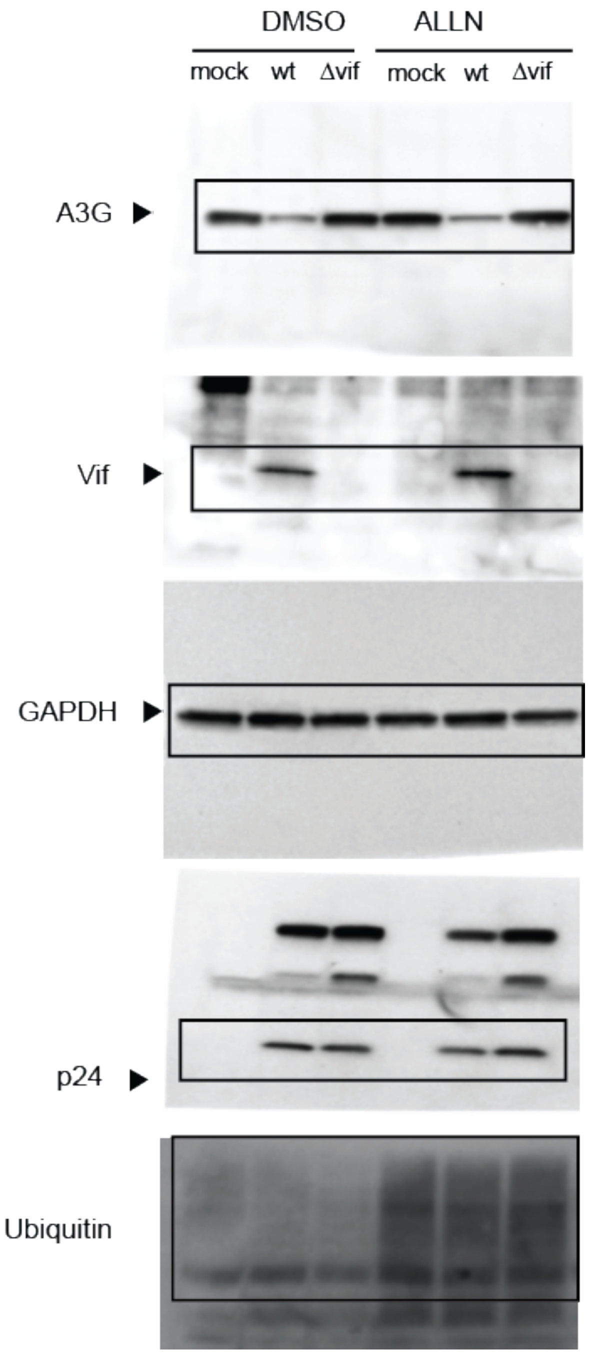

Supplementary Figure S4: Heterologous 5’UTRs do not allow inhibition of A3G translation by Vif. Full-length blots of Figure 4, showing A3G, Vif, with the Actin control that was run on the same gel. Parts that have been cropped are boxed.

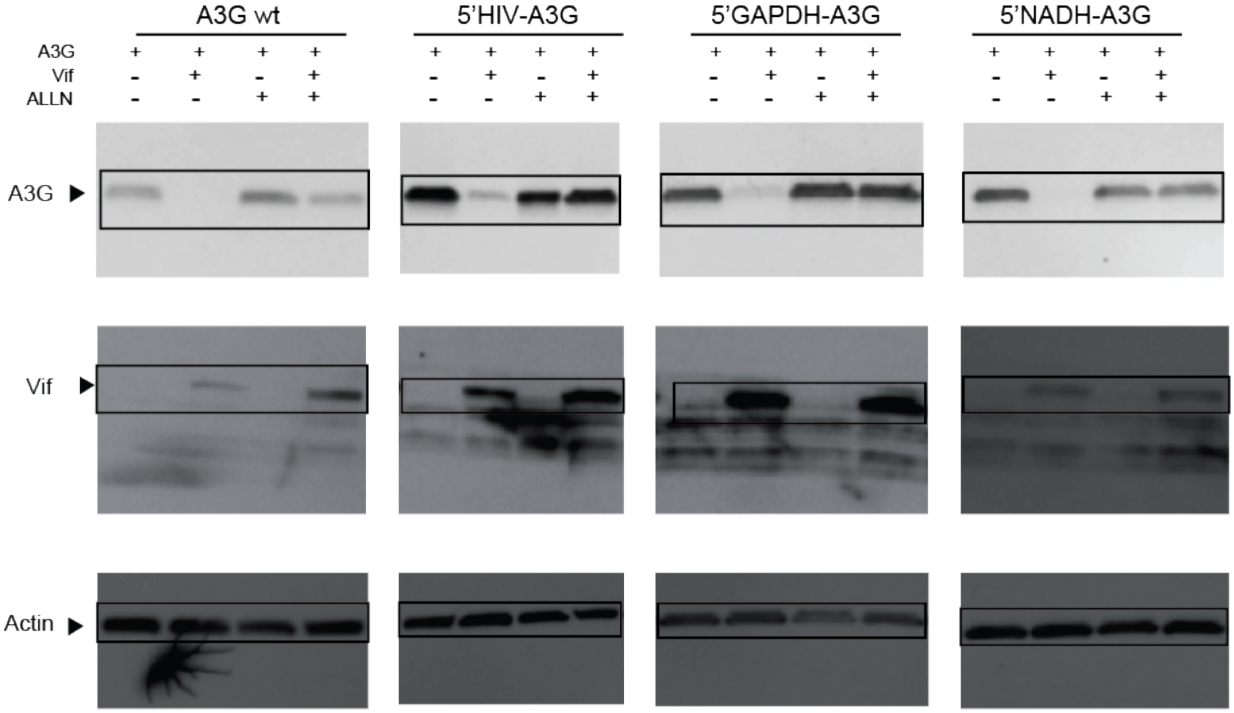

Supplementary Figure S5: Vif requires SL2 and SL3 to impair A3G translation.

Full-length blots of Figure 5 showing A3G, Vif, with the actin control that was run on the same gel. Arrows point to the indicated proteins. Parts that have been cropped are boxed.

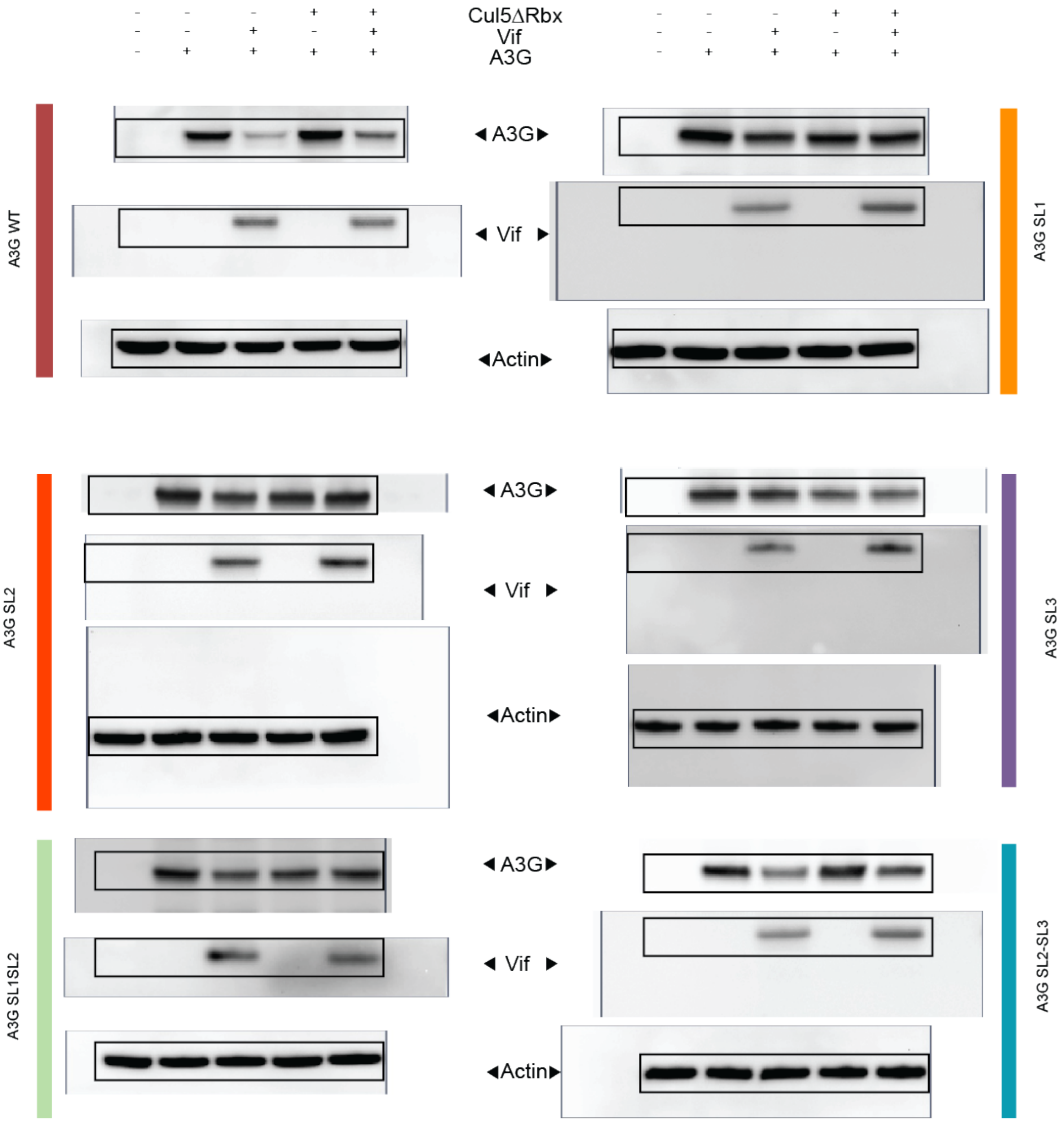

Supplementary Figure S6. Vif K26 residue is required for the translational inhibition of A3G. Full-length blots of Figure 6A and 6B, showing A3G, and Vif (input and IP) with the β-actin control that was run on the same gel. Arrows point to the indicated proteins. Parts that have been cropped are boxed.

(A)

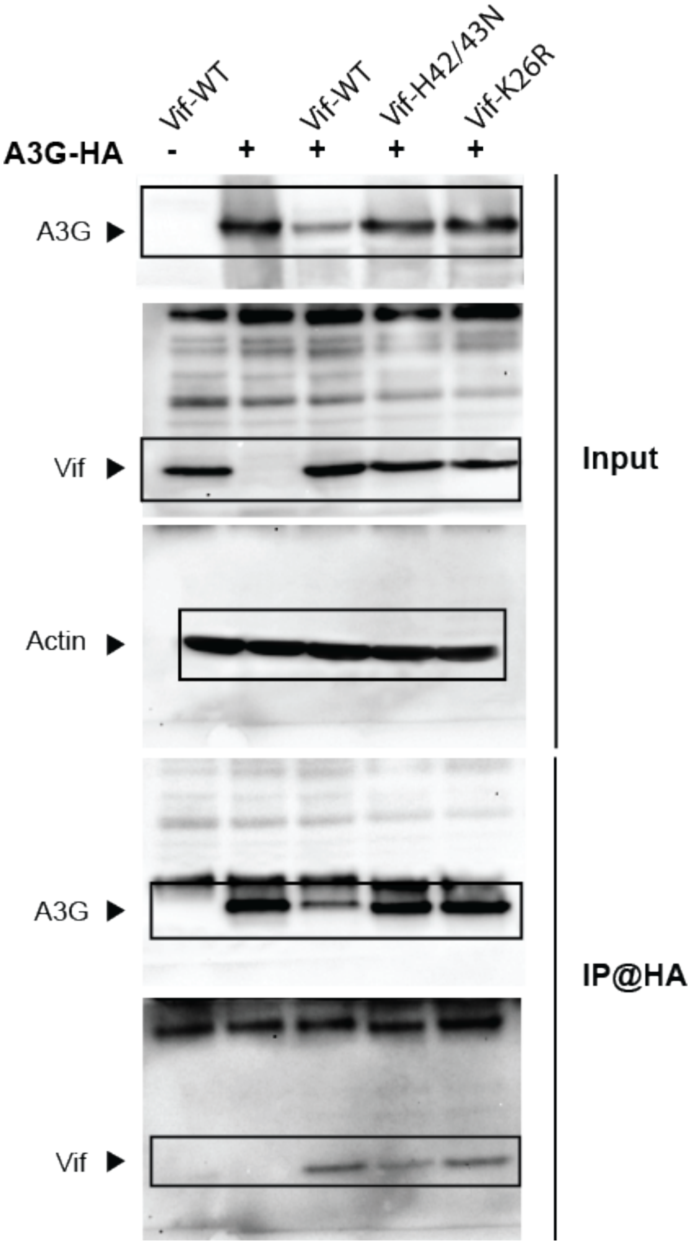

(B)

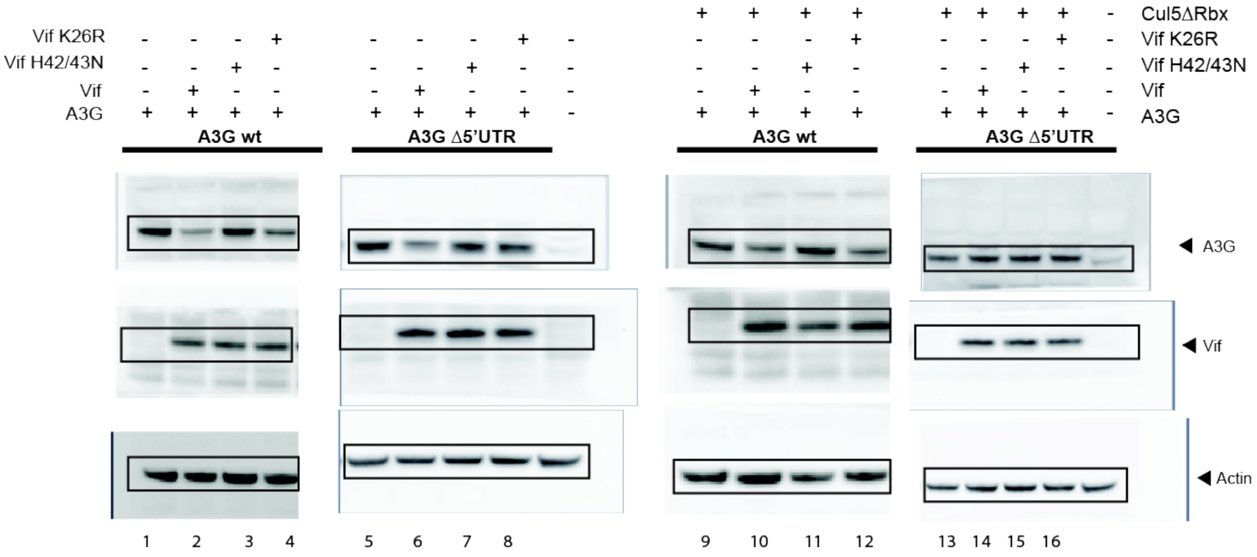
Vif K26R
Vif H42/43N
Vif
A3G
A3G wt
A3G Δ5'UTR
Cul5ΔRbx
A3G
Vif
Actin
1 2 3 4
5 6 7 8
9 10 11 12
13 14 15 16

Supplementary Figure S7: Effect of the inhibition of A3G translation by Vif on A3G packaging and viral infectivity. Full-length blots of Figure 7, showing A3G, and Vif with the β-actin (A) or p24 (B) controls that were run on the same gel. Arrows point to the indicated proteins. Parts that have been cropped are boxed.

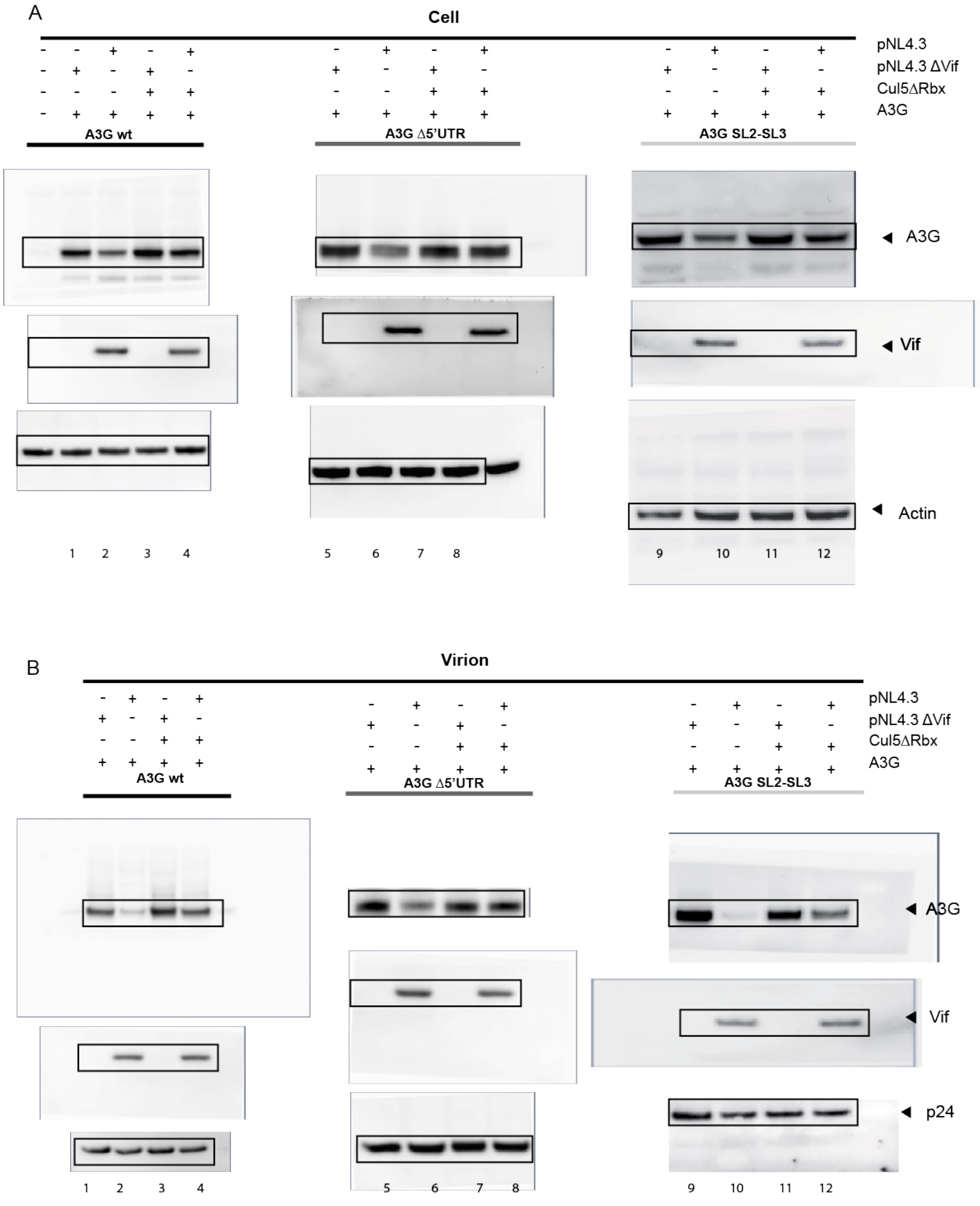

Supplement: Supplementary Information [file srep39507-s1.pdf]
